# Supplementary material for: A single n-type semiconducting polymer-based photo-electrochemical transistor
Source: Nat Commun. 2023 Sep 7;14:5481. doi: 10.1038/s41467-023-41313-7 (PMC10482932; doi:10.1038/s41467-023-41313-7)
Supplement: Supplementary file 1 — Supplementary Information [file 41467_2023_41313_MOESM1_ESM.pdf]

Supplementary information

A single n-type semiconducting polymer-based photo-electrochemical transistor

Victor Druet[1], David Ohayon[1], Christopher E. Petoukhoff[2], Yizhou Zhong[1], Nisreen Alshehri[2,3], Anil Koklu[1], Prem D. Nayak[1], Luca Salvigni[1], Latifah Almulla[1], Jokubas Surgailis[1], Sophie Griggs[4], Iain McCulloch[2,4], Frédéric Laquai[2], and Sahika Inal[1*]

[1] King Abdullah University of Science and Technology (KAUST), Biological and Environmental Science and Engineering Division, Organic Bioelectronics Laboratory, Thuwal 23955-6900, Saudi Arabia

[2] KAUST Solar Center, Physical Science and Engineering Division, Materials Science and Engineering Program, KAUST, Thuwal 23955-6900, Saudi Arabia

[3] Physics and Astronomy Department, College of Sciences, King Saud University, Riyadh 12372, Saudi Arabia

[4] Department of Chemistry, Chemistry Research Laboratory, University of Oxford, Oxford OX1 3TA, UK

[*]Corresponding author: sahika.inal@kaust.edu.sa

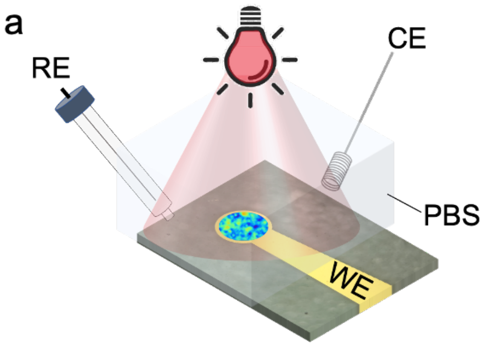

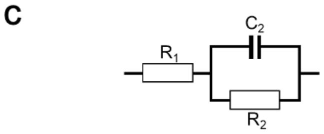

At OCP	Dark	Light
R_1 (kΩ)	8.8	6.8
C_2 (nF)	42	20350
R_2 (MΩ)	136	0.000724

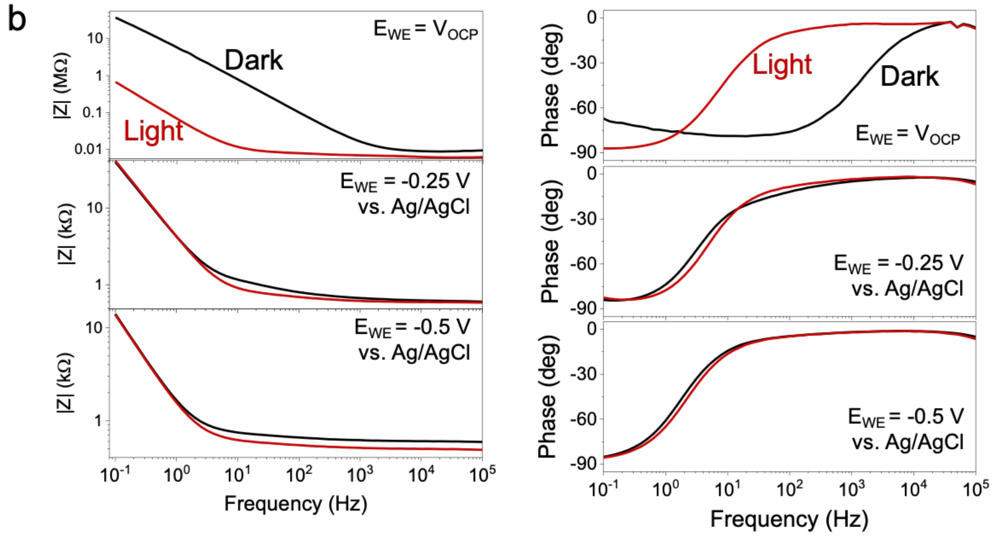

Supplementary Figure 1. Electrochemical impedance spectroscopy in dark and under light (**a**) Schematic of the three-electrode set-up used to investigate the photo-electrochemical properties of a p(C_6NDI-T) coated gold microelectrode (A = 0.196 mm^2) in PBS. RE, CE, and WE are reference, counter, and working electrodes, respectively. (**b**) The impedance magnitude (left) and phase (right) of the p(C_6NDI-T) coated electrode in the dark (black) and under red light illumination (red). The spectra were recorded for three electrochemical states: at OCP (top), at -0.25 V vs. Ag/AgCl (middle), and at -0.5 V vs. Ag/AgCl (bottom). We calculated the impedance magnitude change at 1 Hz due to light at each biasing condition. The impedance magnitude at 1 Hz recorded at OCP is 100 times lower under light than in the dark. On the other hand, once doped, the change is less than 5%. The volumetric capacitance of the doped film was obtained when fitting the spectrum of the biased sample in the dark at -0.5V vs. Ag/AgCl. The estimated error was lower than 2%. (**c**) The Randels circuit fit (top schematic) and the fit parameters underneath. R_1 is the solution resistance, C_1 is the electrode capacitance, and R_2 is the charge transfer resistance.

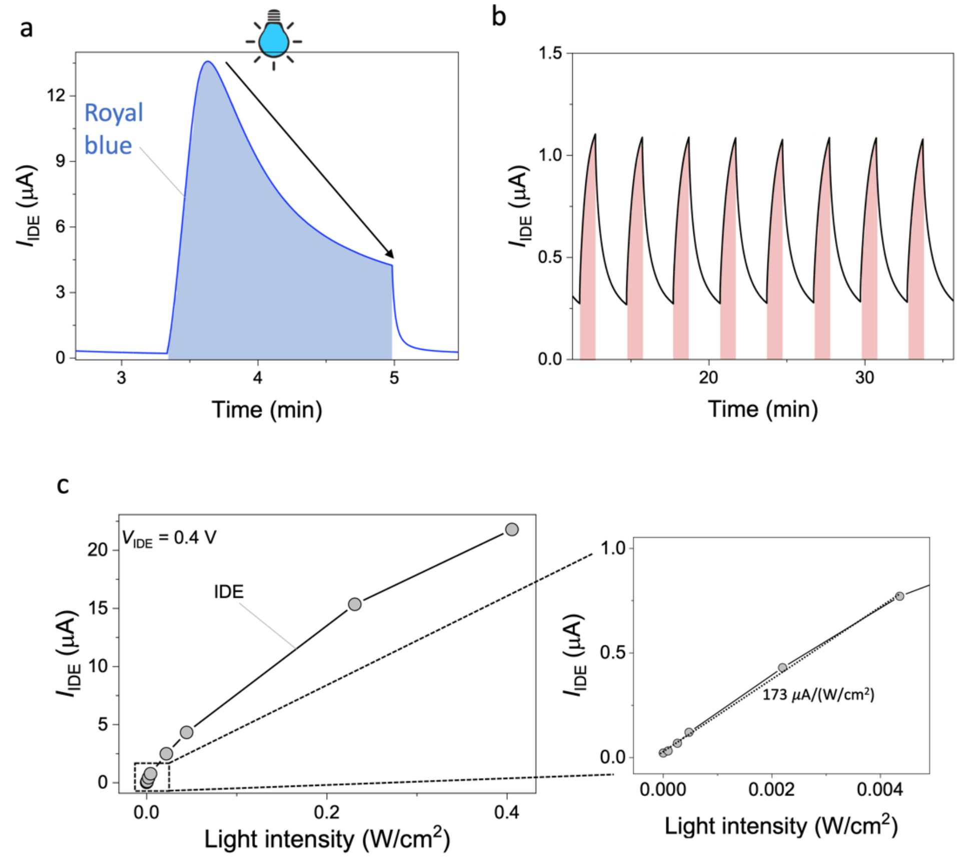

Supplementary Figure 2. The light response of the IDE platform (a) Chronoamperometry experiment (I_{IDE} vs. time) when biasing the p(C_6-NDI-T) IDE at V_{IDE} = 0.4 V and under royal blue (455 nm) illumination. The LED was set at 600 mW/cm^2 and switched on from 3.4 to 5 min. I_{DS} reached a maximum at t= 3.6 min before dropping, although the film was still under blue light illumination. (**b**) Chronoamperometry experiment when biasing the p(C_6-NDI-T) IDE at V_{IDE} = 0.4 V and under deep red (660 nm) pulses. The red LED was set at 150 mW/cm^2 and switched on for 1 min, followed by 2 min dark relaxation. (**c**) The current recorded at V_{IDE} = 0.4 V as a function of light intensity (0 to 406 mW.cm^{-2}), inset shows the response to light intensities lower than 5 mW/cm^2.

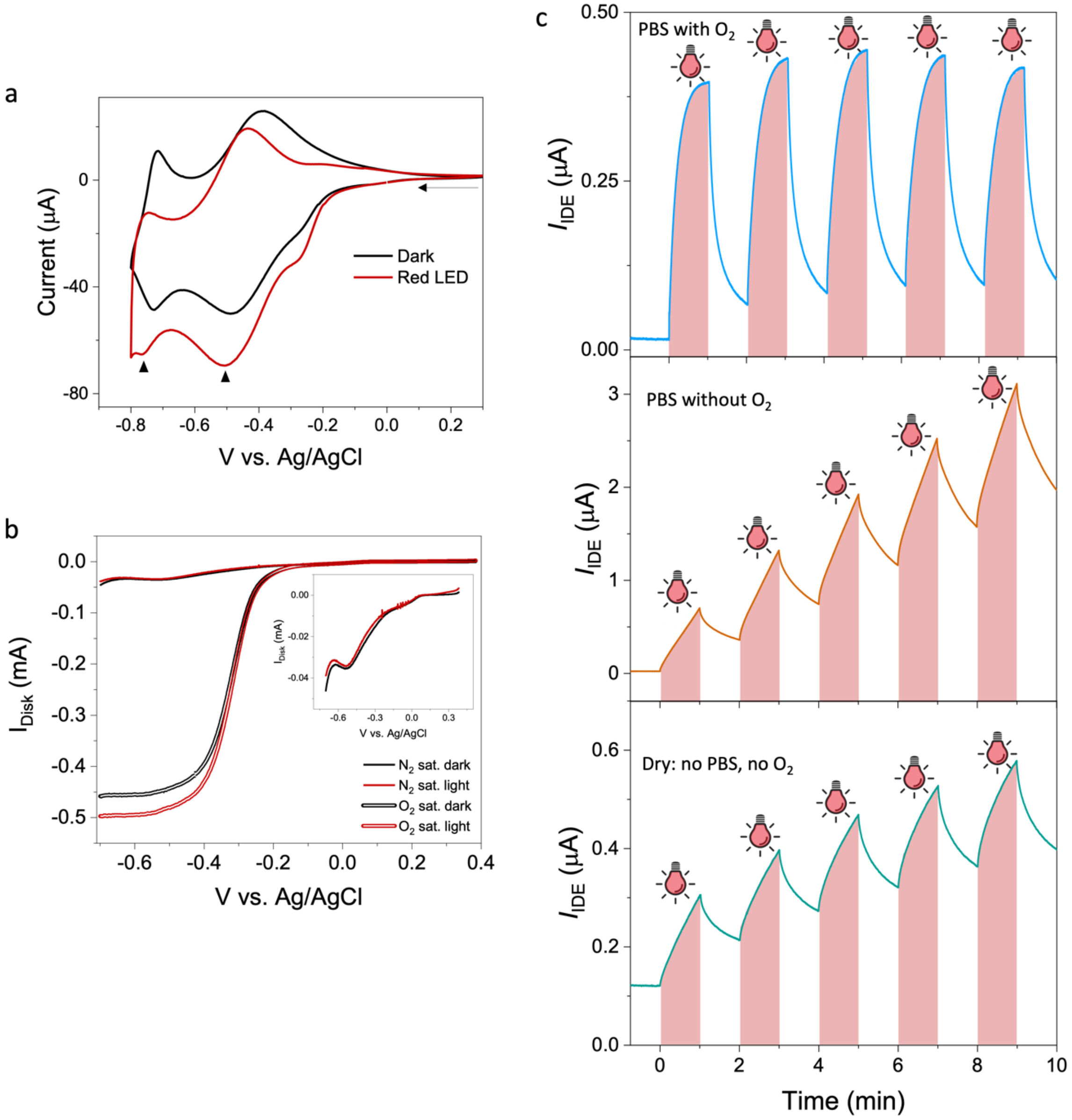

Supplementary Figure 3. The effect of O_2 and water on light-induced current response (a) CV curve of the film measured in the dark and under illumination (660 nm, 406 mW/cm^2). (**b**) Linear sweep voltammetry (LSV) of a p(C_6NDI-T) coated glassy carbon electrode (19.6 mm^2) in a nitrogen (N_2) saturated electrolyte (solid lines) and in O_2 saturated electrolyte (double solid lines), inset zooms in the N_2 saturated LSV curves. The curves were obtained at 1200 rpm enabling convection and thus limiting the influence of the diffusion of redox species on the curves. Upon light illumination, a 10% increase in the reduction current was observed in O_2 saturated electrolyte for potentials lower than -0.4 V vs. Ag/AgCl. **(c)** The change in IDE current during 5 cycles of light pulses (660 nm, 406 mW/cm^2, 1 min) separated by dark conditions (1 min). V_{IDE} = 0.4 V. Measurements were performed in an ambient electrolyte (top panel) and in the absence of O_2 (degassed electrolyte, middle panel) and in the co-absence of O_2 and water (dry film in the glove box, bottom panel). In the ambient electrolyte, a mean photocurrent of 353 nA (I_{light}/I_{dark} = 27) is

generated in one light-dark cycle. Without O_2, a mean photocurrent of 1.2 μA ($I_{light}/I_{dark} = 80$) is generated in one light-dark cycle. Without O_2 and water, a mean photocurrent of only 200 nA ($I_{light}/I_{dark} = 1.7$) is generated in one cycle.

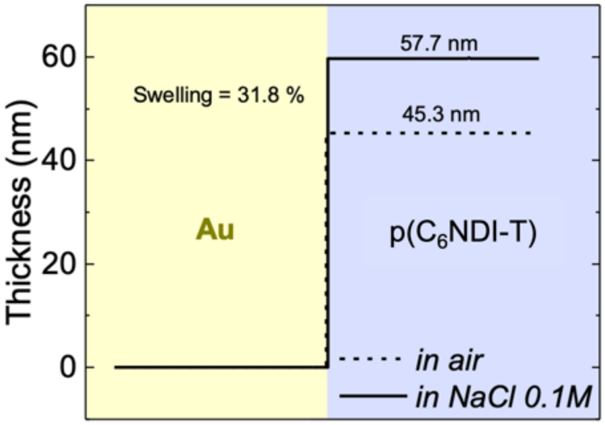

Supplementary Figure 4. QCM-D measurements quantifying film electrolyte uptake. The film swells ca. 31% when exposed to 0.1 M NaCl solution

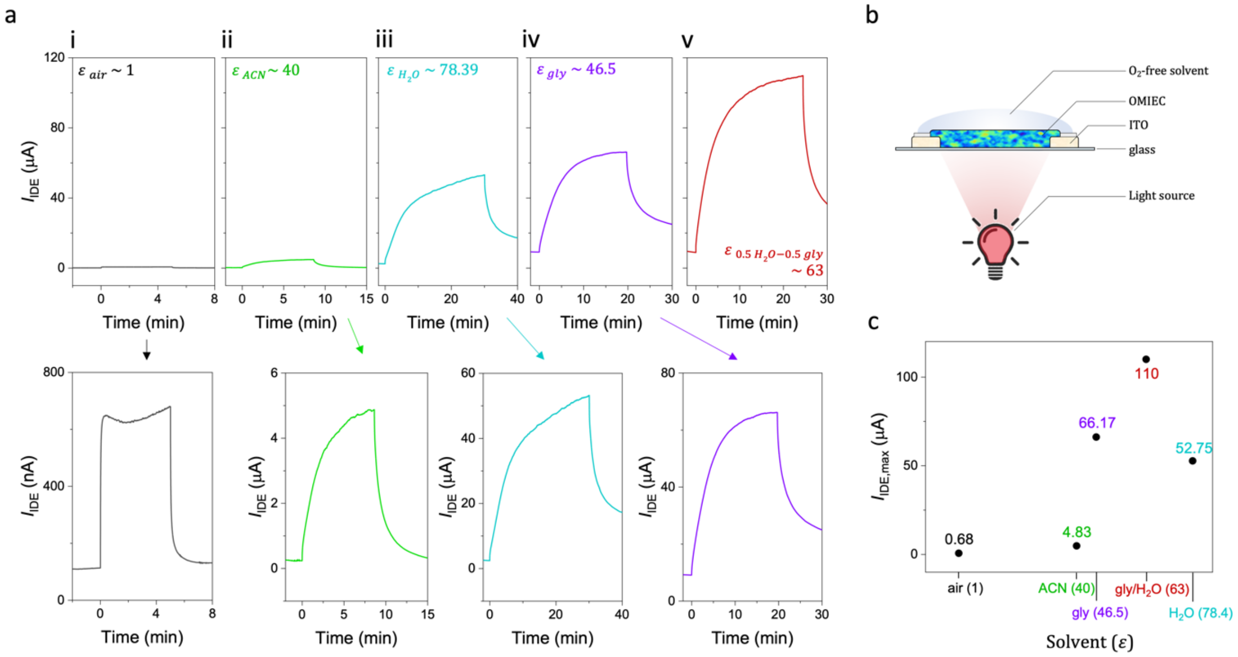

Supplementary Figure 5. The effect of electrolyte dielectric constant on light response (a) Current response of a p(C_6NDI-T) coated IDE electrode (at $V_{IDE} = 0.4$ V) to a light pulse (660 nm, 406 mW/cm^2) when interfacing environments with different dielectric constants: air ($\varepsilon_{air} \sim 1$, **i**), acetonitrile ($\varepsilon_{ACN} \sim 40$, **ii**), water ($\varepsilon_{H2O} \sim 80$, **iii**), glycerol ($\varepsilon_{gly} \sim 46.5$, **iv**), a mixture 50 %v/v water and 50 %v/v glycerol (**v**). **b)** Schematic of the set-up used to collect the photo response. The light

source is placed at the ITO side to eliminate interference from the refractive indices differences. The experiments are performed in an O_2-free environment to accurately compare the nature of the solvent only, considering that O_2 dissolves differently in each solvent **(c)** Maximum current reached upon illumination (upon stabilization or after 25 min of continuous illumination) as a function of the dielectric constant of each environment.

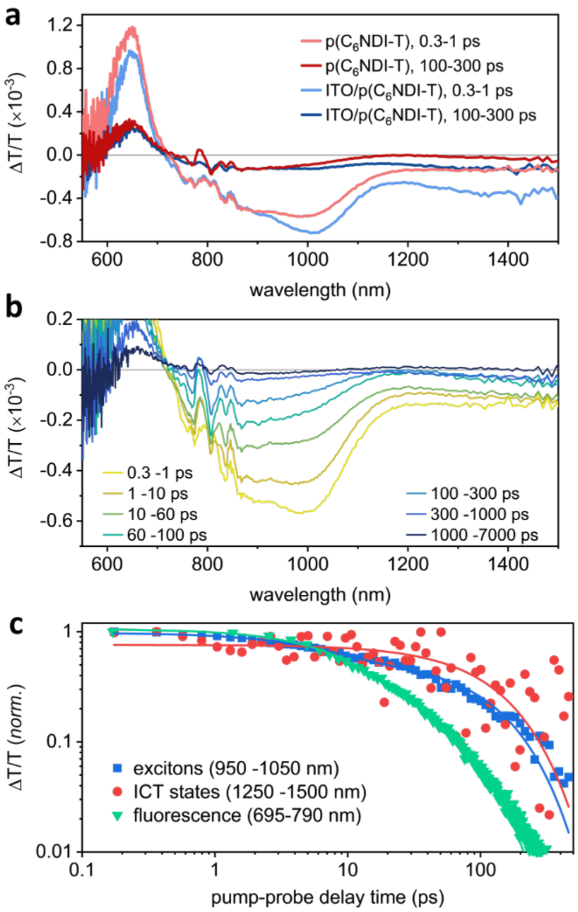

Supplementary Figure 6. TA spectra and fluroescence kinetics (a) TA spectra of intrinsic p(C_6NDI-T) film compared with ITO/p(C_6NDI-T) at early (0.3-1 ps) and late (100-300 ps) timescales. **(b)** TA spectra of intrinsic p(C_6NDI-T) film at different pump-probe delay times. **(c)** Picosecond-nanosecond kinetics of p(C_6NDI-T) film, compared with the time-resolved fluorescence kinetics. Overlaid are double exponential decay fits (see Supplementary Table 1). The fluence for all TA spectra and kinetics was 24 μJ/cm^2, and the fluence for the fluorescence kinetics was 15.6 nJ/cm^2.

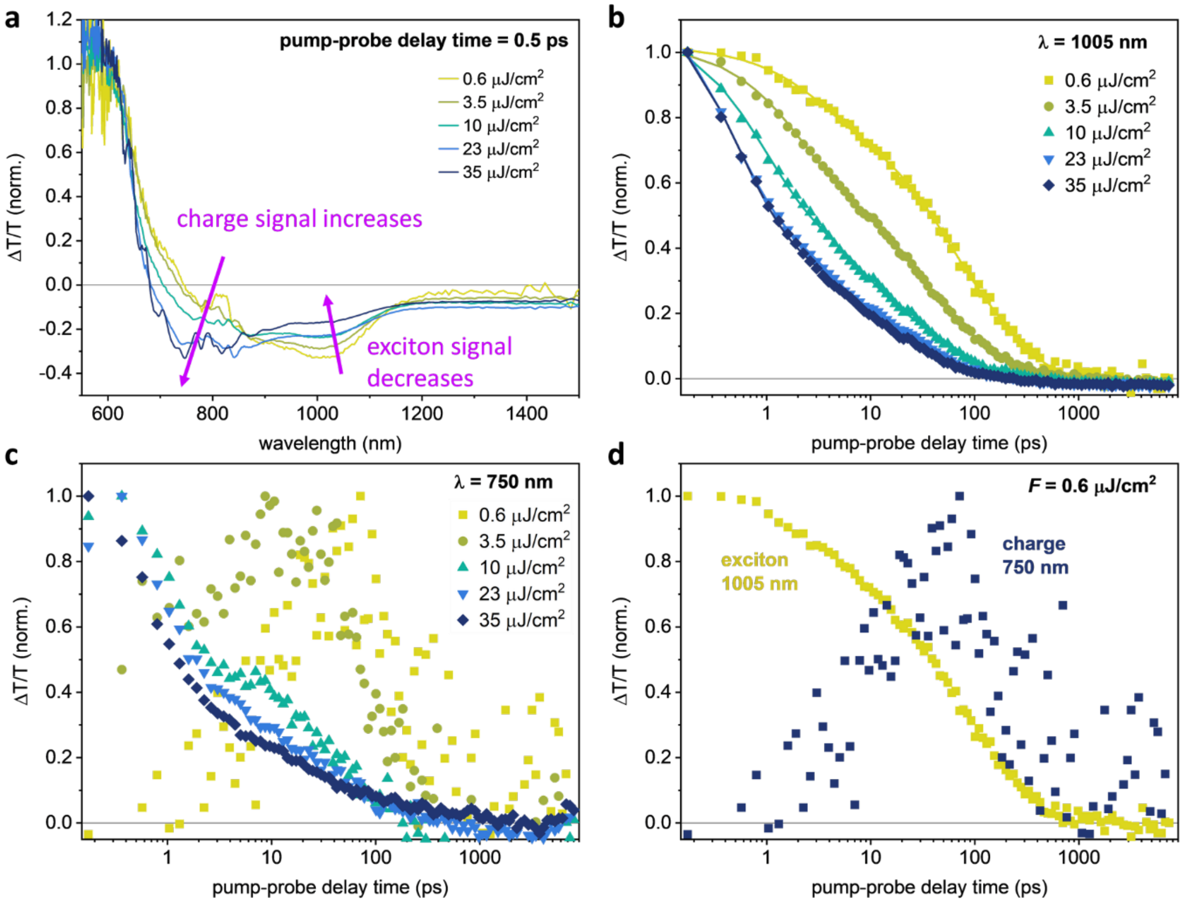

Supplementary Figure 7. TA spectroscopy measurements (a) Normalized TA spectra at different excitation fluences at a pump-probe time delay of 0.5 ps. As the fluence increased, the exciton signal (~1005 nm) decreased in relative intensity while the charge signal (~750 nm) increased. **(b)** TA kinetics extracted from the exciton PIA band (1005 nm). The change in recombination dynamics with increasing fluence is a signature of a second-order (*i.e.*, bimolecular) recombination process. **(c)** TA kinetics extracted from the charge PIA band (750 nm). At low fluence, the charge signal increased with increasing time. This rise time became faster with higher excitation fluence. When the fluence exceeded 10 μJ/cm^2, there were two competing processes for charge generation: exciton-exciton annihilation at lower pump fluences and ultrafast dissociation of excitons upon excitation to higher energy states at higher fluences. **(d)** TA kinetics at low excitation fluence (0.6 μJ/cm^2), showing the rise of the charge signal as the exciton signal decays.

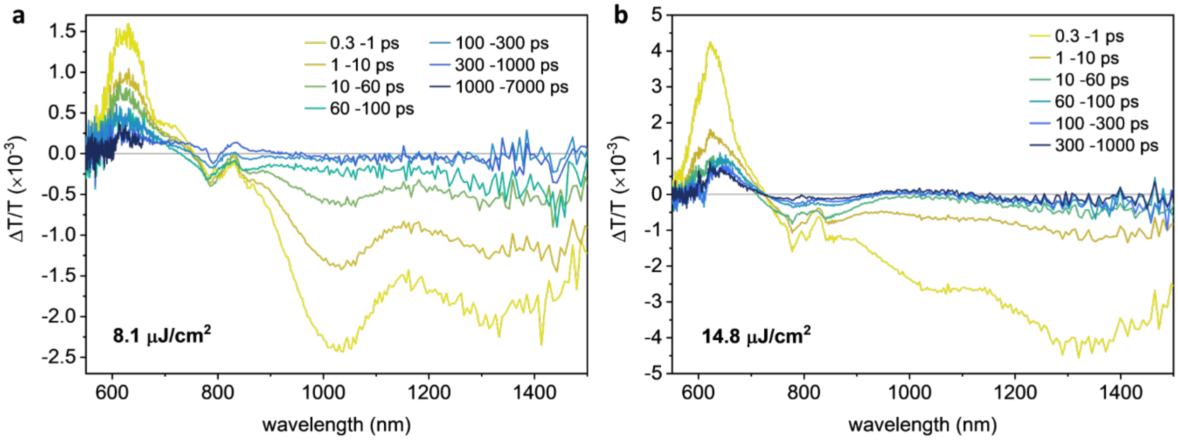

Supplementary Figure 8. TA spectra of ITO/p(C_6NDI-T) for different pump-probe delay times with pump wavelength selected to excite the ground state ICT band resonantly (i.e., 620 nm) at **(a)** 8.1 μJ/cm^2 and **(b)** 14.8 μJ/cm^2 excitation fluences. The ICT PIA band (1250-1500 nm) is more intense relative to the exciton PIA band (900-1100 nm) than in the off-resonant (660 nm) excitation (Figure 3, and Supplementary Figures 6, 7, and 9).

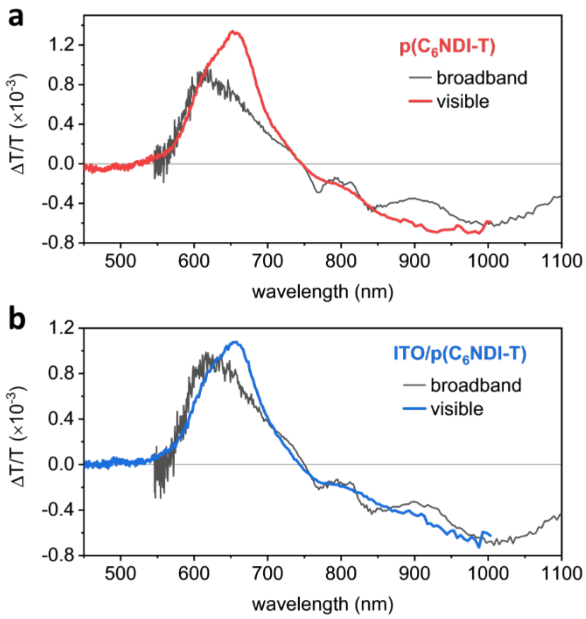

Supplementary Figure 9. Comparison of TA spectra measured using a broadband, InGaAs detector (Hamamatsu G11608-512A) vs. a visible, Si detector (Hamamatsu S8381-512Q) for **(a)** p(C_6NDI-T) and **(b)** ITO/p(C_6NDI-T), at a pump-probe delay time of 0.3-1 ps and 8.1 μJ/cm^2 excitation fluence. The broadband detector used a sapphire crystal with an 800 nm seed wavelength for white light generation (as described in the Methods), whereas the visible detector used a moving CaF_2 crystal with an 1150 nm seed wavelength for white light generation. Spectra obtained from the two detectors did not have significant differences, apart from an increased signal from the GSB and the lack of seed scattering around 800 nm when using the visible detector. No distinctive charge bands were observed in the range of 690-850 nm when using the visible detector.

Supplementary Table 1. Decay parameters obtained from fitting the picosecond-nanosecond TA kinetics (Figure 3d and Supplementary Figure 6c) for different species to the equation: $\Delta T/T = A_1 e^{(-t/\tau_1)} + A_2 e^{(-t/\tau_2)}$ where t is the pump-probe delay time, A_i are the amplitudes of the decay components, and τ_i are the lifetimes of the decay components. The amplitude-averaged lifetime, τ_{ave}, is reported in the final column.

Sample	Species	wavelength (nm)	A_1	τ_1 (ps)	A_2	τ_2 (ps)	τ_{ave} (ps)
p(C_6NDI-T)	excitons	950-1050	0.419	6.36	0.560	127.51	74.1
	ICT	1300-1450	0.762	136.22			103.8
	PL	695-790	0.746	9.86	0.319	61.06	26.8
ITO/ p(C_6NDI-T)	excitons	950-1050	0.380	7.79	0.579	134.43	80.8
	ICT	1250-1500	0.163	4.38	0.752	246.30	185.9
	PL	695-790	0.573	9.86	0.484	65.23	37.2

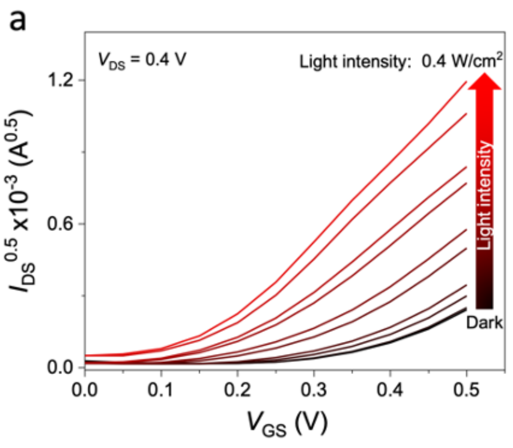

Light Illumination (mW/cm²)	V_{TH} (mV)
0 (Dark)	350
0.02	350
0.14	320
0.31	310
2	280
4.2	260
23	200
46	180
229	150
406	140

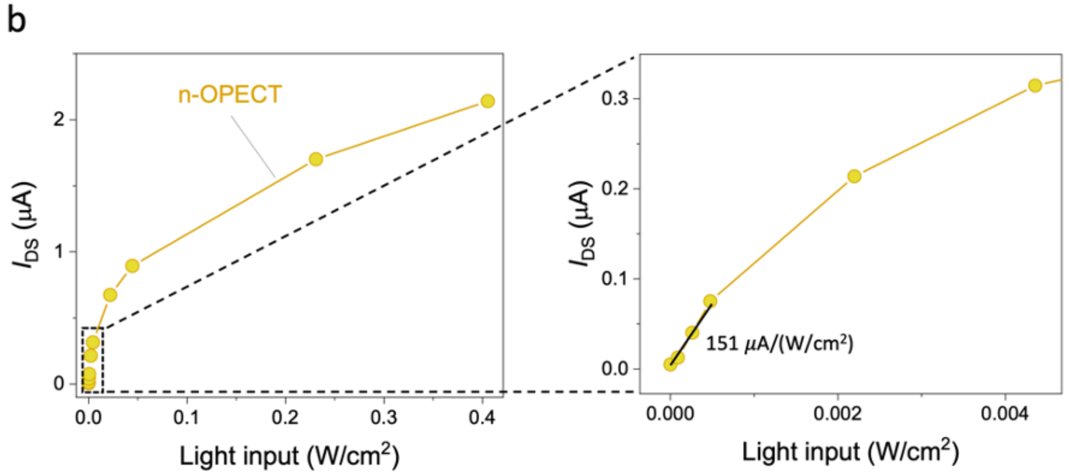

Supplementary Figure 10. The light-induced threshold voltage (V_{TH}) change and the sensitivity of n-OPECT to light (**a**) The square root of I_{DS} vs. V_{GS} reported at V_{DS} = 0.4 V at various light intensities (dark to 406 mW/cm^2). V_{TH} extracted for each condition is displayed in the table on the right-hand side. (**b**) Calibration plot: current response of the n-OPECT (V_{DS} = V_{GS} = 0.4V) as a function of light intensities (0 to 406 mW.cm^{-2}), inset focuses on the response to light intensities lower than 5 mW/cm^2.

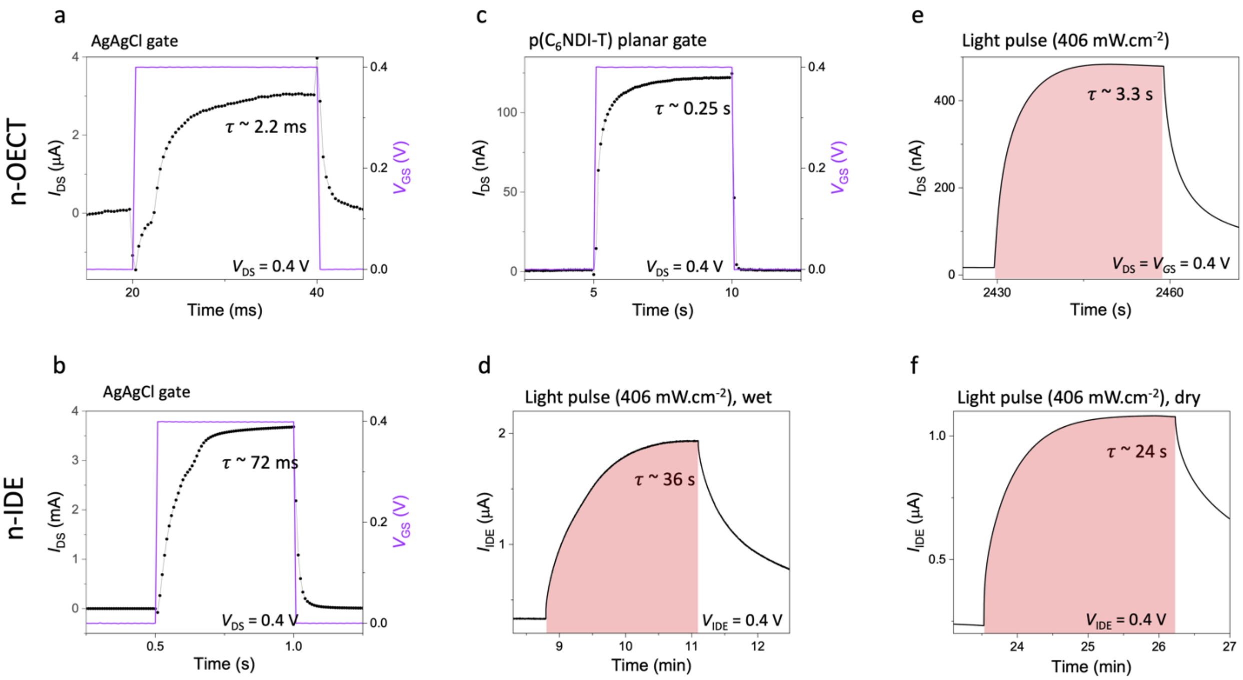

Supplementary Figure 11. Response time characteristics of the OECT and the n-IDE platforms. (a) The current output of **(a)** the OECT channel (A=1000 μm^2) **(b)** the IDE (A = 4 mm^2) used as the channel gated with Ag/AgCl. The current was recorded at V_{DS} = 0.4 V while applying a voltage pulse at the gate with a magnitude of 0.4 V for 20 ms or 0.5 s. **(c)** The transient characteristics of the n-OECT (gate is the p(C_6NDI-T)) monitored at V_{DS} = 0.4 V with V_{GS}= 0.4 V pulse applied for 5 s. The current-time profile of **(d)** the IDE **(e)** the n-OPECT monitored at V_{DS} = V_{GS} = 0.4 V and upon exposure to a light pulse (660 nm, 406 mW.cm^{-2}). The light was kept on until the current stabilized, and its duration is highlighted in red. **(f)** The current-time profile of the IDE in the absence of PBS, monitored under the same conditions as in (d). While the presence of the electrolyte enhances the photocurrent, it also results in a slightly slower transient response. Switching on time of the devices (τ) was estimated by fitting the rising curve (upon gate voltage or light illumination) with an exponential decay function.

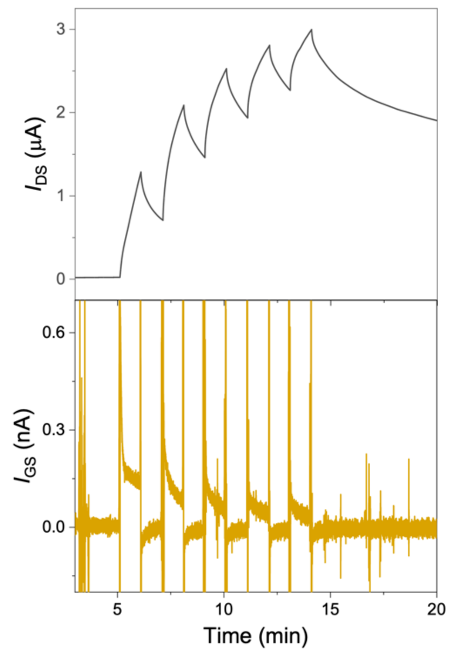

Supplementary Figure 12. The effect of O_2 on light-induced device currents. Current-time profile of n-OPECT upon five consecutive light pulses (660 nm, 406 mW/cm^2) in O_2-free electrolyte. The current was measured at $V_{DS} = V_{GS} = 0.4$ V.

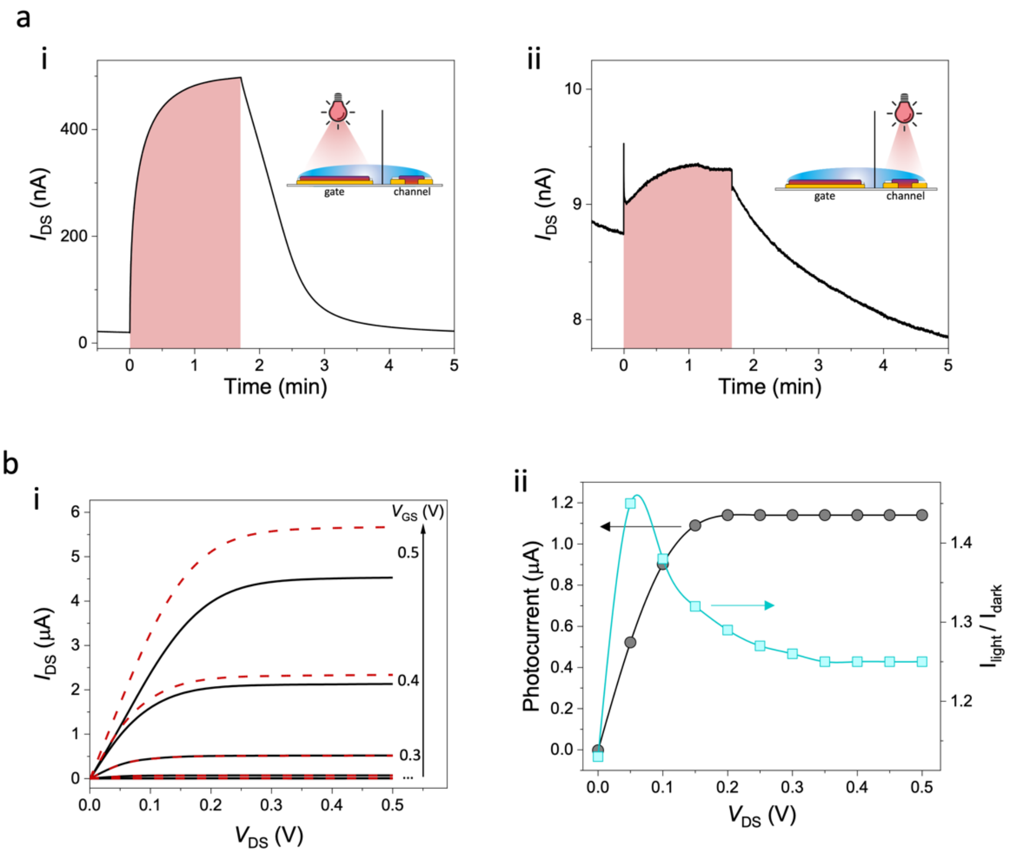

Supplementary Figure 13. The OPECT response with localized light exposure and when the gate electrode is Ag/AgCl. (**a**) The current response (I_{DS}, left Y axis and I_{GS}, right Y axis) of the n-OPECT recorded at $V_{DS} = V_{GS} = 0.4$ V when light (300 μW/cm^2, applied during $0 < t < 1.5$ min) illuminated the gate electrode only **(i)** and the channel only (ii). (**b**)(**i**) Output characteristics of the n-type channel gated with an Ag/AgCl electrode in dark (solid black lines) and under illumination (dotted red lines). (**ii**) Photocurrent and I_{light} /I_{dark} ratio of the Ag/AgCl gated channel. The data were extracted at various V_{DS} while $V_{GS} = 0.5$ V. The maximum I_{light} / I_{dark} ratio is 1.25, which is 30 when using the p(C_6NDI-T) gate (Figure 4b).

Supplementary Table 2. Characteristics and operating conditions of the OPECTs

Publication	Light source		Materials and mechanism					OPECT characteristics						Application
Reference (Year)	Wave length	Power	Photo-active material	Channel material	medium	Photo-conversion mechanism	Device operation	I_{dark}	I_{light}	$\frac{I_{light}}{I_{dark}}$ or $\frac{I_{dark}}{I_{light}}$	$g_{m,\ dark}$	$g_{m,\ light}$	$\frac{g_{m,\ light}}{g_{m,\ dark}}$	
Our work (2023)	660 nm	406 mW. cm^{-2}	$p(C_6NDI\text{-}T)$/ Au gate	$p(C_6NDI\text{-}T)$	PBS only	Potentiometric Photoexcitation of OMIEC	Enhancement	5 nA	2141 nA	428	20 mS.c m^{-1}	775 mS.c m^{-1}	39	Photodetect or, PPG recording, logic gate, neuromorph ic device
[1] (2022)	Unclear, Xe lamp?	-	PEDOT-Fe-MOF/ ITO gate	PEDOT:PSS	PBS	Faradaic photoinduced electron transfer at the p-n interface then collected at the ITO gate	Depletion	-40 µA	-80 µA	2	-	-	-	Pesticide malathion detection
[2] (2023)	425 nm	40 $mW.m^{-2}$	Photo sensitive MOF + TiO_2 NRs / FTO gate	PEDOT:PSS	PBS	Faradaic Photoinduced electrons transferred from the CB of MOF to TiO2 NRs and then to the FTO gate.	Depletion	1.9 mA	0.8 mA	2.4	2.05 mS	2.3 mS	1.12	miRNA-21 detection
[3] (2018)	White light	2000 $mW.m^{-2}$	Poly thiophene / Au gate	PEDOT:PSS	0.1 M NaCl with O_2 required	Faradaic Light enhanced-oxygen reduction reaction	Depletion	-3 mA	-4.8 mA	1.6	0.28 mS	-	-	Opto-logic switch
[4] (2018)	420 nm	0.2 $mW.cm^{-2}$	Cds QDs /ITO gate	PEDOT:PSS	PBS with 0.1M AA	Faradaic Photoexcited electrons are transferred from the CB of Cds QDs to the ITO gate. AA donates an electron to the excited VB of Cds	Depletion	360 µA	60 µA	6	-	-	-	DNA detection<br><br>Heart-type fatty acid protein detection[5]<br><br>Adenosine triphosphat e detection[6]
[7] (2022)	425 nm	5 W lamp	Cds QDs /ITO gate	PEDOT:PSS	PBS with 0.05M AA	Faradaic Photoexcited electrons are transferred from the CB of Cds QDs to the ITO gate. AA donates an electron to the excited VB of Cds	Depletion	1500 µA	750 µA	2	-	-	-	IgG detection
[8] (2022)	White light	0.48 $mW.cm^{-2}$	$CdsIn_2S_4$ /FTO gate	PEDOT:PSS	PBS and 0.01M TEOA	Faradaic Photoexcited electrons are transferred from the CB of $CdsIn_2S_4$ to the FTO gate. TEOA donates an electron to the excited VB of Cds	Depletion	1200 µA	600 µA	2	-	-	-	IgG detection

[9] (2022)	White light	5 W lamp	TiO_2 nanotubes /Ti gate	PEDOT:PSS	Tris-HCl with AA	Faradaic Photoexcited electrons are transferred from the CB of TiO2 to the Ti gate. AA donates an electron to the excited VB	Depletion	525 μA	480 μA	1.09	-	-	-	Membrane protein detection
[10] (2019)	585 nm	0.5 mW. cm^{-2}	J71:ITIC coated onto channel / Au gate	PEDOT:PSS	Ion gel	Faradaic Photo-generated holes transferred to channel, charge separation at the J71:ITIC heterojunction	Depletion	1.25 mA	3.7 mA	2.96	2.5 mS	4 mS	1.6	Flexible image sensor
[11] (2022)	425 nm	16 mW. cm^{-2}	CdS QDs and TiO2/ FTO gate	PEDOT:PSS	1x PBS and AA for photo anode	Faradaic n/p junction reacting with Ascorbic acid and oxygen	Depletion	1.7 mA (Vg= 0V)	0.25 mA (Vg= 0V)	6.8	-	-	-	Logic circuit and metal ions sensor
[12] (2022)	425 nm	20 mW. cm^{-2}	CdS QDs and TiO2 nanotubes / Ti gate	PEDOT:PSS	PBS and MEA	Faradaic Photoexcited electrons are transferred from the CB of Cds QDs to TiO2 and then transferred to the Ti gate. MEA donates an electron to the excited VB of Cds	Depletion	1050 μA	750 μA	1.4	-	-	-	miRNA detection<br><br>Serum alkaline phosphatase detection[13]<br><br>Tear lysozyme detection[14]
[15] (2023)	455 nm	5 W lamp	$ZnIn_2S_4$ photocatalyst heterojunctio n	PEDOT:PSS	PBS and 0.05 M AA	Faradaic Photoexcited electrons are transferred from the CB of $ZnIn_2S_4$ to the FTO gate. AA donates an electron to the excited VB of $ZnIn_2S_4$	Depletion	1200 μA	645 μA	1.9				Sandwich immunocom plex assay for the detection of mouse IgG
[16] (2023)	455 nm	-	PDots on poly(amine) / ITO gate	Dedoped PEDOT:PSS (by amine)	1x PBS and H^+ through pH change	Faradaic Photoinduced holes are transferred from the VB of PDots to the amine HOMO and then to the ITO gate. Photoinduced electrons reduce protons in solution.	Enhancement	-345 μA	-675 μA	2	-	-	-	Urea detection through a pH change
[17] (2023)	365 and 530 nm	365 nm at 3W and 530 nm at 5 mW output	OEG-Spiropyran blend at channel / Pt grid as gate	Blend of pgBTTT with the OEG-SP	0.5 M NaCl	Photoisomerization of spiropyran compound modulating the channel doping state	Depletion	33.5 μA (SP form, 1st cycle)	26 μA (MC form, 1st cycle)	1.3	-	-	-	Optical switch

Abbreviations:
CB: conduction band; VB: valence band; ITO: indium tin oxide; MOF: metal-organic framework; NRs: nanorods; FTO: fluorine-doped tin oxide; QDs: quantum dots; AA: ascorbic acid; MEA: monoethanol amine; PDots: polymer dots; Pt: platinum; $CdIn_2Sn_4$: cadmium indium sulfide; $ZnIn_2S_4$: zinc indium sulfide; TiO_2: titanium oxide; OEG-SP: oligo(ethylene glycol)-spiropyran

Supplementary Table 3. Details of the different light cycles used when operating the n-OPECT synapse.

Light cycle #	Δt_{light} (ms)	Δt_{dark} (ms)	$\Delta t_{light} / \Delta t_{dark}$	Light cycle Frequency (Hz)
1	100	5000	50	0.2
2	100	2000	20	0.48
3	100	1000	10	0.91
4	100	500	5	1.67
5	100	200	2	3.33
6	100	100	1	5
7	100	50	0.5	6.67

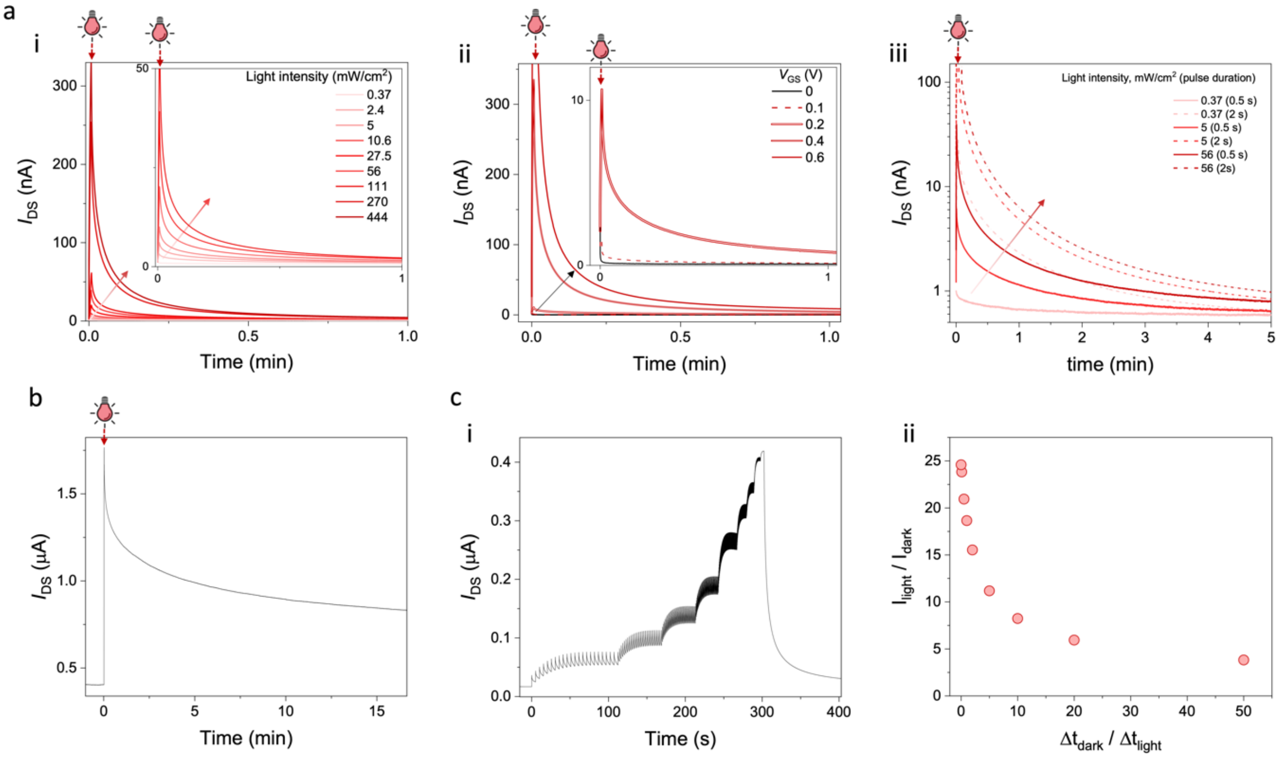

Supplementary Figure 14. Detailed memory behavior of OPECT. (a-i) Current response recorded at $V_{DS} = V_{GS} = 0.4$ V to a single light pulse of 500 ms long (660 nm, initiated at t = 0 s). Higher pulse intensity promotes a larger spiking current and better current retention. The arrow

indicates the increase in light intensity. **(a-ii)** Current response to a single light pulse (660 nm, 500 ms long, 444 mW/cm^2, initiated at t = 0 s) monitored at different gate voltages. Higher gate voltage promotes a higher spiking current and a longer retention of the current, indicated by the arrow direction. **(a-iii)** Current response recorded at $V_{DS} = V_{GS} = 0.4$ V to a single light pulse of three different intensities (660 nm, 0.37 – 5 – 56 mW/cm^2) applied for 500 ms or 2 s for each intensity. Higher pulse intensity and longer pulse duration together promote a higher spiking current and longer current retention. (**b**) Current response to a single light pulse (660 nm, 444 mW/cm^2 for 500 ms, initiated at t = 0s). Measurement was performed in an O_2-free electrolyte, inducing current retention after light pulses exceeding 10^3 s, realizing much longer time retention of the synaptic information. (**c-i**) Current response to light pulse cycles of increasing frequencies. Each light cycle has an identical pulse (660 nm, 444 mW/cm^2 for 100 ms) followed by various relaxation times (spanning from 5 s to 10 ms). Each light cycle frequency is repeated until a stable current is reached. (**c-ii**) I_{light}/I_{dark} response (recorded after the values stabilized after each light pulse cycle) vs. the ratio of the time spent in the dark and the time under illumination.

Supplementary References

1 Ding, L. *et al.* Turning on High-Sensitive Organic Electrochemical Transistor-Based Photoelectrochemical-Type Sensor over Modulation of Fe-MOF by PEDOT. *Advanced Functional Materials* **32**, 2202735 (2022).

2 Gao, G. *et al.* Functional Metal–Organic Frameworks for Maximizing Transconductance of Organic Photoelectrochemical Transistor at Zero Gate Bias and Biological Interfacing Application. *Advanced Functional Materials*, 2300580 (2023).

3 Kolodziejczyk, B., Ng, C. H., Strakosas, X., Malliaras, G. G. & Winther-Jensen, B. Light sensors and opto-logic gates based on organic electrochemical transistors. *Materials Horizons* **5**, 93-98 (2018).

4 Song, J. *et al.* Organic Photo-Electrochemical Transistor-Based Biosensor: A Proof-of-Concept Study toward Highly Sensitive DNA Detection. *Advanced healthcare materials* **7**, 1800536 (2018).

5 Shi, Z. *et al.* Ascorbic acid-mediated organic photoelectrochemical transistor sensing strategy for highly sensitive detection of heart-type fatty acid binding protein. *Biosensors and Bioelectronics* **201**, 113958 (2022).

6 Lu, M.-J. *et al.* Tuning the Surface Molecular Charge of Organic Photoelectrochemical Transistors with Significantly Improved Signal Resolution: A General Strategy toward Sensitive Bioanalysis. *ACS sensors* **7**, 2788-2794 (2022).

7 Hu, J. *et al.* Multifunctional Hydrogel Hybrid-Gated Organic Photoelectrochemical Transistor for Biosensing. *Advanced Functional Materials*, 2109046 (2022).

8 Li, C. J. *et al.* Biomolecules-Incorporated Metal-Organic Frameworks Gated Light-Sensitive Organic Photoelectrochemical Transistor for Biodetection. *Advanced Functional Materials*, 2211277 (2022).

9 Li, Z. *et al.* Light-Fueled Organic Photoelectrochemical Transistor for Probing Membrane Protein in an H-Cell. *Advanced Materials Interfaces* **9**, 2102040 (2022).

10 Yan, Y. *et al.* High-performance low-voltage flexible photodetector arrays based on all-solid-state organic electrochemical transistors for photosensing and imaging. *ACS applied materials & interfaces* **11**, 20214-20224 (2019).

11 Xu, Y. T. *et al.* Bipolar modulation of the ionic circuit for generic organic photoelectrochemical transistor logic and sensor. *Advanced Optical Materials* **10**, 2102687 (2022).
12 Gao, G. *et al.* Hybridization chain reaction for regulating surface capacitance of organic photoelectrochemical transistor toward sensitive miRNA detection. *Biosensors and Bioelectronics* **209**, 114224 (2022).
13 Ban, R. *et al.* Biological modulating organic photoelectrochemical transistor through in situ enzymatic engineering of photoactive gate for sensitive detection of serum alkaline phosphatase. *Biosensors and Bioelectronics* **218**, 114752 (2022).
14 Li, Z. *et al.* Organic photoelectrochemical transistor detection of tear lysozyme. *Sensors & Diagnostics* **1**, 294-300 (2022).
15 Zhu, Y. *et al.* Bioderived establishment of three-dimensional type-I Ag2S/ZnIn2S4 heterojunction for high-efficacy organic photoelectrochemical transistor biomolecular detection. *Analytica Chimica Acta* **1240**, 340757 (2023).
16 Yuan, C. *et al.* Polymer Dot-Gated Accumulation-Type Organic Photoelectrochemical Transistor for Urea Biosensing. *ACS sensors* **8**, 1835-1840 (2023).
17 Turetta, N. *et al.* A photo-responsive organic electrochemical transistor. *Journal of Materials Chemistry C* (2023).
